# Walking asymmetry and its relation to patient-reported and performance-based outcome measures in individuals with unilateral lower limb loss

**DOI:** 10.1080/23335432.2022.2142160

**Published:** 2022-11-22

**Authors:** Christopher K. Wong, Emily E. Vandervort, Kayla M. Moran, Carly M. Adler, Stanford T. Chihuri, Gregory A. Youdan

**Affiliations:** a Department of Rehabilitation and Regenerative Medicine, Columbia University Irving Medical Center, New York, NY, USA; b Program in Physical Therapy, Columbia University, New York, NY, USA; c School of Public Health, Columbia University Irving Medical Center, New York, NY, USA; d Teacher’s College, Columbia University, New York, NY, USA

**Keywords:** Gait, symmetry, balance, amputation, prostheses, patient-reported outcomes

## Abstract

Gait asymmetry persists for most people after lower limb amputation and is associated with slower walking speeds. However, the relationship between gait asymmetry and patient-reported function remains unclear because they are not commonly assessed together. The purpose of this study was to determine relationships between gait asymmetries in people with lower limb loss and (1) patient-reported outcomes and (2) performance-based prosthetic functional measures. This cross-sectional analysis included nine people with unilateral limb loss aged 48.2 ± 13.1 years of mixed amputation etiology. Patient-reported outcomes included the Prosthetic Evaluation Questionnaire mobility subscale and Activities-specific Balance Confidence scale. Performance outcomes included the Berg Balance Scale and the 30-second sit-to-stand test. Walking performance measures included the 2-Minute Walk Test, during which APDM Opal sensors recorded spatiotemporal gait parameters, and daily step-counts from StepWatch4 activity monitors. The study found that the most asymmetric gait symmetry ratios (prosthetic-limb divided by intact-limb) could be attributed to prosthetic foot dorsiflexion-plantarflexion and rotation motion limitations: prosthetic-limb trailing double support (0.789 ± 0.052), toe-off (0.760 ± 0.068) and toe-out angle (0.653 ± 0.256). Single limb stance, and stance and swing phase durations were most strongly associated with balance and walking performance measures. Notably, no symmetry ratio was significantly associated with patient-reported prosthetic function (unadjusted Pearson correlation coefficients *r* < 0.50, *P* > 0.05). More gait symmetry was associated with better balance and walking performance but had no significant relationship with patient-reported function. Although achieving gait symmetry after lower limb loss is a common walking goal, symmetry was unrelated to the perception of functional mobility for people with lower limb loss.

## Introduction

For people with lower limb loss, gait symmetry remains an elusive rehabilitation goal that can be difficult to achieve despite years of walking with a prosthetic leg, although the impact of gait symmetry on patients’ perception of mobility is unclear (Carse et al. 2020). Spatiotemporal asymmetries associated with gait speed, such as longer duration intact-limb stance phase and shorter duration prosthetic-limb swing phase, are common in both transtibial and transfemoral prosthetic walking (Nolan et al. 2003). Asymmetric timing, however, does not consistently result in spatiotemporal asymmetries commonly observed in clinical practice, such as short intact-limb step length (Roerdink et al. 2012). Direction of asymmetry varies amongst individuals and the magnitude of asymmetry varies with walking speed; thus, step length alone can be insufficient for evaluating gait asymmetry in people with limb loss (Roerdink et al. 2012). Recurring kinematic gait asymmetries, such as limited prosthetic hip joint extension in late stance and reduced knee flexion in early swing, are observed in more than 80% of individuals with lower limb loss (Carse et al. 2020). Limited hip motion at least partially explains asymmetric kinetic measures such as limited hip extension moment and power generation, while the movement limitations of prosthetic componentry may explain ankle and knee moment and power generation asymmetry (Carse et al. 2020). The most consistent gait asymmetry may be uneven weight bearing, marked by more intact-limb weight acceptance than the prosthetic leg (Nolan et al. 2003). Whether because of discomfort, prosthetic alignment, muscle weakness, or reduced proprioception, uneven weight bearing leads to asymmetry that increases with faster walking speed in people using either transtibial or transfemoral prostheses (Nolan et al. 2003).

Gait asymmetry has been suggested as a factor in decreased physical function, poor gait efficiency, and the development of secondary orthopedic conditions (Kline et al. 2019), but the evidence remains unclear. Physical function as measured by walking speed has not been consistently associated with gait symmetry (Nolan et al. 2003). Increased walking speeds reduce temporal asymmetry but increase loading asymmetry (Nolan et al. 2003). The suggestion that gait efficiency measured by energy consumption increases with more symmetric gait was observed in one cohort of people with non-vascular amputation (Darter et al. 2013) but not supported by other studies in older people with transtibial amputations of mixed causes (Yeung et al. 2012) or younger military people with traumatic transfemoral amputations (Mahon et al. 2019). Evidence is also mixed for the suggestion that gait asymmetries in people with limb loss lead to secondary health issues such as knee osteoarthritis and low back pain (Lloyd et al. 2010; Devan et al. 2017). People using leg prostheses exhibit potential kinematic and kinetic risk factors for osteoarthritis (Lloyd et al. 2010), though the actual incidence of knee arthritis in people with limb loss is low compared to matched controls (Welke et al. 2019). However, compensatory trunk movements during gait (Gaffney et al. 2018) may explain the high prevalence of low back pain in people with transfemoral amputations (Devan et al. 2017). Likewise, swing phase knee and hip flexion asymmetry may factor into trips and falls and could partially explain the high fall-related injury rates in people with limb loss (Wong et al. 2016a).

Prosthetic componentry and physiotherapy approaches can reduce gait asymmetries for people with lower limb loss (Highsmith et al. 2016; Cutti et al. 2018; Houdijk et al. 2018). For people with transfemoral amputations, microprocessor knees can reduce stance phase duration and loading asymmetry to provide foot clearance and loading response symmetry comparable to people with transtibial amputations (Cutti et al. 2018). For people with transtibial amputations, energy storing feet reduce step length asymmetry compared to nonenergy storing solid ankle cushion heel feet by significantly increasing late stance push off (Houdijk et al. 2018). Prosthetic componentry such as energy storing feet and microprocessor knees can overcome common gait asymmetries by enhancing motions that otherwise require significant muscle output to minimize asymmetry, such as increased residual-limb hip extension (Silverman et al. 2008). While symmetry outcomes after hip strengthening programs have not been specifically explored, prosthetic rehabilitation approaches generally improve gait compared to baseline, though randomized control trial evidence remains sparse (Highsmith et al. 2016). In small cohorts of <10 subjects, increased over-ground or treadmill walking with or without augmentative feedback has been shown to improve symmetry (Highsmith et al. 2016). Integrating walking training that incorporates visual feedback with a powered prosthetic knee may further reduce stance time asymmetry (Brandt and Huang 2019). Despite advances in prosthetic componentry and physiotherapy to maximize gait symmetry, most people with lower limb loss continue to demonstrate asymmetric gait patterns (Carse et al. 2020).

Gait asymmetry could be considered an anticipated normal after limb loss given the loss of lower limb length, weight, and joint and muscle function after amputation. Prosthetic and anatomic limb length are rarely equal (Friberg 1984). Running legs are built longer than the intact limb to accommodate for the compressive spring action in stance phase but then remain longer in swing phase potentially leading to compensatory deviations (Beck et al. 2016). Walking legs may be aligned shorter than the intact limb to accommodate for lack of active swing phase ankle-toe dorsiflexion (Wong and Edelstein 2019). Emerging technology for microprocessor ankles in advanced prosthetic feet provides swing phase ankle dorsiflexion, but no commercially available prosthetic feet reproduce swing phase toe dorsiflexion to be symmetrical with that typically occurring at anatomically intact metatarsal phalangeal joints (Laferrier and Gailey 2010). The loss of limb weight is not typically matched symmetrically by the prosthesis. However, when the prosthesis is made to match sound limb weight and inertia, the lack of active residual-limb musculature and the altered sensations of wielding the prosthesis result in increased asymmetry (Mattes et al. 2000). Even in static standing, with or without a prosthesis, people with limb loss stand asymmetrically with center of gravity biased towards the intact-limb (Ku et al. 2014). Some suggest that asymmetries can be beneficial compensations for lost limb function that enhance overall function for people living with limb loss (Hak et al. 2014). A degree of gait asymmetry may well be normal.

While persistent gait asymmetry for people with lower limb loss can influence walking speed, the relationship between gait asymmetry and patient-reported perceptions of function remains unclear (Kark and Simmons 2011). Gait speed was associated with patient satisfaction in the prosthesis, walking, and well-being; however, gait deviations were not significantly associated with functional outcome (Kark and Simmons 2011). Gait symmetry measures have not commonly been examined with respect to patient-reported functional outcome and were not a significant factor in patient-reported physical adjustment after amputation in a recent review (Luza et al. 2020). If asymmetry has no strong relationship to subjective assessment of functional mobility by people with lower limb loss, then effort during physiotherapy may best be focused on balance and functional mobility goals rather than achieving perfect gait symmetry. The purpose of this study was to determine relationships between gait asymmetries and patient-reported outcomes and performance-based measures of prosthetic walking ability in people with unilateral limb loss. Based on past literature, the hypothesis was that gait asymmetry would be associated with walking ability but not patient-reported outcomes.

## Methods

This study was a cross-sectional analysis of a prospective cohort approved by the participating medical center Institutional Review Board #AAAR7523. The study sample was anticipated to reflect the characteristics of the local population: adults 18–70 years old of any sex or race with unilateral transtibial or transfemoral limb loss, of any amputation cause, who had completed their initial prosthetic training and were living at home were eligible to be included. Subjects who could not understand the study description for any reason; had a diagnosed medical condition affecting balance including blindness, vestibular disorders, traumatic brain injury, hemiplegia/paraplegia; or had a medical condition making exercise dangerous such as unstable angina or uncontrolled hypertension were excluded. The sample size for the 1-year funding period was similar to other gait symmetry studies (Silverman et al. 2008; Houdijk et al. 2018). To detect a significant effect size, if present, with sufficient statistical power (80%), the minimum required sample size was calculated for with *r* = 0.70 using the formula correlations (Bujang and Baharum 2016).

Sample size $N={\left(\frac{Z_{\alpha }+Z_{\beta }}{C}\right)}^{2}$ = 10

where the standard normal deviate for *α *=$\;Z_{\alpha }=1.96$, the standard normal deviate for β  = $Z_{\beta }$ = 0.84, and C = 0.5 × ln[(1 +*r*)/(1−*r*)] = 0.8673.

### Procedures

After providing informed written consent to participate, subjects were screened for the ability to use their prosthesis to walk independently with or without walking aid – with those unable to walk at least 3 m excluded. Subjects then completed the study questionnaire; underwent physical balance and walking ability assessments using wearable sensors; and a step count monitor was attached to the prosthesis for remote monitoring. All data were obtained by the research investigators after group training on all aspects of the assessments.

Questionnaire data included demographic information, amputation history including prosthetic type, and walking ability level classified by Houghton score (Wong et al. 2016b). Patient-reported outcome measures included the Prosthetic Evaluation Questionnaire mobility subscale (PEQ-MS) and Activities-specific Balance Confidence scale (ABC). The ABC gauges perceived confidence when performing 16 common activities; the PEQ-MS assesses perceived ability in 12 functional tasks including navigating stairs and escalators (Miller et al. 2001). The PEQ-MS and ABC have both demonstrated good test–retest reliability and excellent internal validity in people with limb loss (Miller et al. 2001).

Physical assessment of balance and walking was performed without the use of walking aids and was augmented with instrumented wearable sensors. Balance ability was measured with the Berg Balance Scale, a 14-task clinical assessment of static and dynamic balance that has strong validity upon Rasch analysis and excellent reliability in people with limb loss (Wong et al. 2013; Wong 2014). Walking speed was determined with the 2-Minute Walk Test (2MWT), a standard and reliable measure for people with limb loss (Brooks et al. 2002). While each subject performed the 2MWT, six APDM Opal v2 sensors calculated spatiotemporal gait parameters. Two sensors each were placed on the subjects’ shoes, one sensor each was placed over the sternum and lumbar spine with separate harnesses, and two sensors placed on the posterior wrists. The sensors integrate accelerometer, gyroscope, and magnetometer data (Selles et al. 2005). Data were transmitted wirelessly to a laptop and processed using APDM Mobility Lab software with a proprietary algorithm, to provide precise spatiotemporal measurement of gait performance (https://www.apdm.com/mobility/) (Selles et al. 2005). Kinematic data included stride length, cadence, stance and swing time, and single and double support time defined as the time spent with one or two limbs on the ground. Data also included midswing foot elevation and deviation from the forward path; and toe-out angle in the transverse plane during midstance and toe-off angle in the sagittal plane during late stance. Daily step counts were obtained with a StepWatch4 Modus Health Step Activity Monitor (https://modushealth.com) strapped just above the lateral prosthetic ankle as an objective measure of physical activity, defined as the daily average of the first full week of 24-hour/day collection post-evaluation (Hordacre et al. 2014). A full week consisted of 7 continuous days to capture all weekend and weekday days. Each day included the midnight to midnight 24-hour data collection period with steps recorded for each day. To ensure completeness of data, steps were recorded every day during the study. Subjects were directed to wear the StepWatch4 during their usual activities without removing it from the prosthetic leg and return the following week for data download. Accelerometer data were downloaded and stored using the laptop base station and processed with StepWatch4 software, with each prosthetic step multiplied by 2 to represent total steps taken, as previously validated (Hordacre et al. 2014). General lower limb strength was assessed with the 30-second sit-to-stand test, which incorporates endurance, provides continuous data, and has good reliability and moderate criterion-related validity (Jones et al. 1999).

### Statistical analysis

Descriptive statistics for demographic and clinical characteristics were tabulated. Symmetry ratio is a unitless measure equivalent to the quotient of prosthetic-limb divided by intact-limb raw data, such that perfect symmetry =1. Symmetry ratio was the preferred equation for expressing gait symmetry, as opposed to a symmetry index that provides percent symmetry with positive or negative sign indicating direction, because the symmetry ratio quotient itself indicates direction of asymmetry allowing correlation with other continuous data (Patterson et al. 2010). Symmetry ratios <1 indicated prosthetic-limb less than intact-limb; symmetry ratios >1 indicated prosthetic-limb more than intact-limb. Symmetry ratios included step length and cadence; stance and swing percent of gait duration; single limb stance, double support (with prosthetic leg leading and prosthetic leg trailing) percent of gait duration; midswing elevation and circumduction distance; and toe-off and toe-out angles. Because gait asymmetries can occur in normal walking, with 95% confidence intervals ranging from 4% to 20% in time-based measures (Herzog et al. 1989), any asymmetry >20% was noted. Symmetry ratios of people with transtibial and transfemoral amputations were compared and analyzed together if not statistically different (*P* >0.05) (Cutti et al. 2018). Assumptions of normality were tested and upheld for all symmetry ratios and outcome measures (Shapiro–Wilk *P* >0.05, skew < 2.0, kurtosis < 7.0). Thus, Pearson correlation coefficients were calculated to determine the strength of association of the average gait symmetry ratios with patient-reported and clinical measures. Correlations were considered moderate when unadjusted coefficients ranged from 0.50 to 0.70 and good when coefficients were greater than 0.70 (Mukaka 2009). Statistical analysis completed using SPSS v.26 for Mac (IBM Corporation).

## Results

Twelve subjects were recruited, 11 enrolled and one subject never attended, one subject’s APDM data was lost due to software malfunction, and one subject moved too slowly for the inertial sensors and step activity monitors to capture. The nine participants (eight men, one woman) with symmetry data for analysis were aged 48.2 ± 13.1 years with 4.8 ± 3.9 years since amputation. Amputation causes included peripheral artery disease (4), trauma (3), and other medical (2), with six transtibial and three transfemoral amputations. Six subjects were classified as Medicare Functional Classification K3 level and three were K2 level walkers (Table 1). Two of three people with transfemoral amputations used microprocessor prostheses. Symmetry ratios between people with transtibial and transfemoral amputations were not statistically different (*P* > 0.05) and thus analyzed together (Cutti et al. 2018).

**Table 1.** Subject information.

Age	Level	Amputation cause	Years since amputation	Prosthesis type	K Level	BBS	ABC (%)	2MWT (m)	PEQ-MS
60	TTA	Dysvascular	6	1C62 Triton, Otto Bock	K2	48	81	125	38
41	TTA	Other Medical	1	Taleo, Otto Bock	K3	56	96	147	48
63	TFA	Other Medical	6	CLeg + CWalk, Otto Bock	K3	51	82	95	30
62	TFA	Dysvascular	10	CLeg4 + 1C60 Triton, Otto Bock	K3	43	83	94	43
55	TTA	Dysvascular	2	1C62 Triton, Otto Bock	K3	50	77	94	39
26	TTA	Trauma	2	Pro-Flex XC, Össur	K3	55	89	165	42
37	TTA	Trauma	1	Re-flex Rotate, Össur	K3	55	74	140	31
33	TFA	Trauma	11	3R80 + 1C60 Triton, Otto Bock	K2	56	96	165	45
51	TTA	Dysvascular	8	Runway, Freedom Innovation	K2	56	64	120	39

Abbreviations: TFA = transfemoral amputation, TTA = transtibial amputation; ABC = Activities-specific Balance Confidence scale, BBS = Berg Balance Scale, 2MWT = two-minute walk test (walking speed is m walked on the 2MWT divided by 120 s), PEQ-MS = Prosthetic Evaluation Questionnaire Mobility Subscale

Asymmetric gait variables that differed by >20% between prosthetic- and intact-limbs were terminal double support with prosthetic-limb trailing 0.789 ± 0.052, toe-off angle 0.760 ±0.068, and toe-out angle 0.653 ±0.256; all other gait symmetry ratios demonstrated <10% average asymmetry (Table 2). Walking performance (2MWT; daily step count) was most associated with gait symmetry in duration of stance phase (*r* = 0.813, *P* < 0.01; *r* = 0.746, *P* = 0.021), swing phase (*r* =−0.846, *P* < 0.01; −0.770, *P* = 0.015), and single limb stance (*r* = 0.845, *P* < 0.01; *r* = 0.750, *P* = 0.02). Berg Balance Scale performance was associated with single limb stance symmetry (*r* = 0.779, *P* = 0.013), while strength on the 30-second sit-to-stand test was associated with toe-off angle symmetry (*r* = 0.790, *P* = 0.011). Patient-reported function on the PEQ-MS was not significantly associated with any symmetry measures (*r* < 0.50, *P* > 0.05); ABC was only associated with terminal double support (*r* = 0.777, *P* = 0.014) (Table 3). In secondary analysis, 2MWT was associated with daily step activity (*r* = 0.692, *P* =0.018) and Berg Balance Scale score (*r* = 0.756, *P* = 0.007), but not PEQ-MS (*r* = 0.288, *P* = 0.391).

**Table 2.** Symmetry ratios.

Gait measure	Symmetry ratio (SD)	Symmetry ratio range
Cadence Rate	1.000 (0.001)	1.00–1.00
Stride Length	0.983 (0.050)	0.89–1.06
Stance %	0.947 (0.036)	0.90–1.00
Swing %	1.090 (0.067)	1.00–1.19
Double Support % – Prosthesis Leading	0.998 (0.003)	0.99–1.00
Double Support % – Prosthesis Trailing	0.789 (0.156)	0.52–0.97
Single Limb Stance %	0.921 (0.056)	0.84–1.01
Elevation in Midswing	0.934 (0.896)	0.13–2.34
Circumduction Distance in Midswing	1.014 (0.729)	0.33–2.38
Toe-Out Angle in Midstance	0.653 (0.767)	0.27–2.26
Toe-Off Angle in Late Stance	0.760 (0.203)	0.47–1.20

Symmetry ratios <1 indicate asymmetry with prosthetic-limb less than intact-limb. Symmetry ratios >1 indicate asymmetry with prosthetic-limb more than intact-limb. % = percent of gait cycle

**Table 3.** Significant relationships (unadjusted Pearson’s correlation coefficients) between select symmetry ratios and outcome measures.

Symmetry ratio	Walking			Balance		Strength
	2MWT	Steps/Day	PEQ-MS	BBS	ABC	30s STS
Stance %	0.813 *	0.746 *	0.393	0.771 *	0.349	0.131
Swing %	−0.846 *	−0.770 *	−0.311	−0.799 *	−0.344	−0.191
Double Support % – Prosthesis Trailing	0.413	0.102	0.403	−0.093	0.777 *	0.398
Single Limb Stance %	0.845 *	0.775 *	0.349	0.779 *	0.381	0.187
Toe-Off Angle in Late Stance	0.170	0.393	0.306	−0.257	0.203	0.790 *

* = significant at *p* < 0.05

Abbreviations: ABC = Activities-specific Balance Confidence scale, BBS = Berg Balance Scale, PEQ-MS = Prosthetics Evaluation Questionnaire Mobility Subscale, 2MWT = 2-Minute Walk Test, 30STS = 30-second Sit-to-Stand, % = percent of gait cycle

## Discussion

This study determined relationships between gait asymmetries and patient-reported and performance-based measures of prosthetic walking ability in people with unilateral limb loss. An association between gait asymmetry and walking ability was expected based on past research, while the comparative absence of research linking gait asymmetry with patient-reported outcomes (Kark and Simmons 2011) suggested a strong association between gait symmetry and patient-reported outcomes would not be found. Gait symmetry positively correlated with balance and walking performance measured by the Berg Balance Scale, 2MWT distance and daily step count. However, gait symmetry measures including those that demonstrated >20% asymmetry did not significantly correlate with patient-reported outcomes of prosthetic function as measured by the PEQ-MS. For the study participants, whose walking ability ranged from K2 independent-household to K3 independent-community level (Table 1) (Wong et al. 2016b), walking symmetrically had no significant relationship with their perception of overall mobility using their prosthesis. This finding was consistent with a cross-sectional study of high performing people with limb loss that found self-perceived function and attitude were the strongest correlates, and asymmetric gait deviations were unimportant, with respect to patient satisfaction (Kark and Simmons 2011). Terminal double support with prosthetic-limb trailing was the only symmetry measure associated with balance confidence. Some gait asymmetries may be functional compensations that serve a purpose (Hak et al. 2014), such as attaining walking speeds similar to able-bodied friends and family or sufficient for safe community access (Wong et al. 2016b).

Study findings that gait symmetry was positively associated with walking speed were consistent with the findings of others (Nolan et al. 2003; Roerdink et al. 2012). Single limb stance and swing phase duration symmetry in particular were related to faster walking speeds and greater average daily steps. That more symmetric gait parallels improved walking speed and energy expenditure has been observed (Darter et al. 2013) but also contradicted (Yeung et al. 2012; Mahon et al. 2019). Some common asymmetries observed in this study, such as reduced single limb stance and terminal double support time with prosthetic-limb trailing, were consistent with reduced double support attributed to limited prosthetic ankle dorsiflexion that inhibits advance of the body over the foot (Carse et al. 2020). Other asymmetric gait deviations observed in the current study such as toe-off and toe-out angles (Table 2) were likely related to the limited prosthetic ankle dorsiflexion and plantar flexion during push off. Limited ankle dorsiflexion in late stance can lead the individual to progress over the foot positioned in more toe-out to shorten the lever length of the foot in the sagittal plane while lack of active ankle plantarflexion limits normal toe-off angle once the heel comes off the ground. Both were unrelated to either physical assessment or patient-reported outcomes. Overall, gait asymmetry may not be easily characterized (Roerdink et al. 2012) due to the wide variation among people with limb loss (Carse et al. 2020), residual limb length and muscular fixations, prosthetic componentry and rehabilitation, and pre-existing musculoskeletal limitations and co-morbidities (Wong et al. 2020), as well as selected walking speeds (Roerdink et al. 2012).

Stance and swing percent of gait cycle, and specifically single limb stance asymmetry, were most strongly correlated to balance and walking performance, and in turn overall physical activity measured by daily steps taken (Table 3). General lower limb strength required to support the body against gravity in the closed chain, assessed by the 30-second sit-to-stand test, was associated with toe-off angle. Toe-off angle represented the ability to maintain balance during stance phase specifically during push off after the heel rises off the ground, which can determine how far the opposite swing leg can advance. Strength may play a factor in gait asymmetry of people with lower limb loss, particularly hip extension strength, and may help optimize terminal double support symmetry by improving advancement over the prosthetic foot (Silverman et al. 2008; Roerdink et al. 2012).

Specific swing phase gait deviations were not associated with any outcome measure. Foot elevation in midswing was one common gait deviation potentially related to trips and falls that was not among the most asymmetric in the current study (Schafer et al. 2018). This may have been because in the earlier study with mixed amputation levels, 9 of the 10 people with transfemoral amputations included in the study used mechanical knee units (Schafer et al. 2018). In the current study, two of three people with transfemoral prostheses used microprocessor knee units which have been shown to provide a gait pattern similar to people with transtibial prostheses (Cutti et al. 2018). The symmetry ratios for both elevation in midswing and circumduction had large standard deviations indicating wide variation within the sample that could account for the lack of significant associations, potentially related to prosthetic knee type (Cutti et al. 2018), residual limb length, and related hip flexion strength (Jaegers et al. 1995). The only gait asymmetry associated with a patient-reported outcome was terminal double support with prosthetic-limb trailing which was related to balance confidence. Inability to advance over the prosthetic foot can make the person feel as if they are falling backwards, especially when walking up inclines. Some asymmetry appears likely to persist even years after training but may not be a significant factor in patient satisfaction (Kark and Simmons 2011).

For the person living with limb loss, persistent gait asymmetry may be a functional compensation that becomes accepted over time. Subjects in this study averaged nearly 5 years since amputation (Table 1). An explanation for why symmetry ratios were associated with balance and walking performance but not patient-reported functional mobility in this study was that performance-based and patient-reported walking and balance ability measures were themselves not strongly correlated. This finding was consistent with a larger study that found patient-reported prosthetic mobility assessed with the PEQ-MS was only moderately associated in bivariate analysis with 2MWT, with <44% of the value of one explained by the other (Wong et al. 2016b). Multiple variable analysis found patient-reported prosthetic mobility and timed walking ability were best explained by different sets of variables, though the analysis did not include gait symmetry ratios (Wong et al. 2020). Gait symmetry may be important to people with limb loss in ways not captured by outcome measures in the literature to date. Anecdotes from study subjects provide some insight. For instance, one subject reported that he did not want to walk with a limp because he felt that it made him feel vulnerable within the community. Symmetry may influence other patient-reported measures, such as body image or self-efficacy, that address a different component of a person’s experience after limb loss. One example may be the Amputee Body Image Scale, which has been reliability and validity tested and associated with performance-based mobility (Desrochers et al. 2019), or other measures of self-esteem or self-efficacy (Miller et al. 2018).

The primary limitation of this study was the small sample size and recruitment from a single urban area. While the sample size was similar to gait symmetry studies in people with lower limb loss (Silverman et al. 2008; Houdijk et al. 2018), a larger sample representing rural, suburban, and urban areas from different geographic areas may have yielded different results, given potentially different physical barriers within the community. For instance, the ABC includes using escalators, which may not be equally common in all communities. A related limitation was the combined analysis of both people with transtibial and transfemoral amputations and different K levels. Effort was taken, however, to first test assumptions of normality and second determine whether significant gait symmetry differences existed between subjects with different amputation levels before analysis, with the lack of significant differences in this study consistent with previous research that documents comparable gait measures with microprocessor knees (Cutti et al. 2018). While all people with lower limb loss are affected by loss of the intact musculoskeletal system and sensation provided by the foot, future research with larger samples would be required to clarify the relationship between gait asymmetries and patient-reported outcomes for different age, sex, or K level subgroups. Another limitation was that APDM sensors have not been validated for people with limb loss, although the technology has been validated in other patient populations and similar accelerometer technology has been validated for people with limb loss (Selles et al. 2005).

## Conclusion

Gait symmetry was associated with performance-based measures of balance and walking but had minimal relationship with patient-reported outcomes. The finding that gait symmetry was not related to patient-reported outcomes does not mean that people with limb loss do not care about symmetry nor that prosthetists and physiotherapists should not work towards gait symmetry. Physiotherapy and prosthetic interventions to improve symmetry of foot clearance in midstance and advancement over the prosthetic foot during single limb support could improve walking performance. However, the results of this study showed that a more symmetrical gait was not associated with a patient perception of better functional mobility. Taking into account both objective performance-based measures and subjective patient-reported outcomes may be necessary to obtain a complete assessment of rehabilitation outcomes for people with lower limb loss.

## Data Availability

Due to the nature of this research, participants of this study did not agree for their data to be shared publicly, so supporting data is not available.
